# Recent Advances in Scaffolds for Guided Bone Regeneration

**DOI:** 10.3390/biomimetics9030153

**Published:** 2024-03-01

**Authors:** Theodoros-Filippos Valamvanos, Xanthippi Dereka, Hector Katifelis, Maria Gazouli, Nefeli Lagopati

**Affiliations:** 1 Laboratory of Biology, Department of Basic Medical Sciences, Medical School, National and Kapodistrian University of Athens, 11527 Athens, Greece; 2 Department of Periodontology, School of Dentistry, National and Kapodistrian University of Athens, 11527 Athens, Greece; xderek@dent.uoa.gr; 3 School of Science and Technology, Hellenic Open University, 26335 Patra, Greece; 4 Greece Biomedical Research Foundation, Academy of Athens, 11527 Athens, Greece

**Keywords:** scaffolds, nanomaterials, alveolar bone defect, tissue engineering, regenerative medicine, alveolar bone regeneration, guided bone regeneration

## Abstract

The rehabilitation of alveolar bone defects of moderate to severe size is often challenging. Currently, the therapeutic approaches used include, among others, the guided bone regeneration technique combined with various bone grafts. Although these techniques are widely applied, several limitations and complications have been reported such as morbidity, suboptimal graft/membrane resorption rate, low structural integrity, and dimensional stability. Thus, the development of biomimetic scaffolds with tailor-made characteristics that can modulate cell and tissue interaction may be a promising tool. This article presents a critical consideration in scaffold’s design and development while also providing information on various fabrication methods of these nanosystems. Their utilization as delivery systems will also be mentioned.

## 1. Introduction

One of the most significant areas of the human body, in terms of function and aesthetic, is the oral and maxillofacial region. Due to the anatomical complexity and the tissue variability, the restoration of alveolar and maxillofacial bone defects occurring from inflammation, periodontal disease, neoplastic pathology, or trauma is challenging to achieve [1,2]. Bone has a limited healing capacity which is inadequate to regenerate larger size defects [3,4]. Furthermore, the use of titanium dental implants is considered a predictable treatment option for partial and full edentulism, provided that there is an adequate bone amount at the recipient site for the successful placement of implants in the prosthodontically-driven ideal position [5]. Subsequently, the augmentation of the defected sites followed by the restoration with dental implants requires the advancement of bone tissue engineering (BTE). BTE is a rapidly growing field, which develops biofunctional tissues that can substitute the diseased or damaged ones [6]. Guided tissue regeneration (GTR) is a principle introduced in the mid-1980s. According to this principle, it is possible to achieve the regeneration of a certain type of tissue, when the defect is populated with cells capable of regenerating this particular type of lost tissue during the healing phase. Based on this principle, the guided bone regeneration (GBR) concept was developed [7,8,9]. Dahlin et al. (1988) introduced GBR as a therapeutic modality to achieve bone regeneration [10]. This concept utilizes barrier membranes (resorbable/non-resorbable) to prevent the ingrowth of certain cell types, including rapidly proliferating epithelium and connective tissue, hence promoting the growth of slower-growing cells that are responsible for bone formation [9,11,12,13,14]. In many instances, GBR is combined with bone grafting procedures/materials. Currently, the transplantation of autogenous bone from an intra-oral or extra-oral donor site is considered the gold standard method due to the low immunogenicity and disease transmission risk [15]. Even though anatomical areas, including the mandibular symphysis and maxillary tuberosity, may provide excellent autologous bone grafting sites, the harvesting capacity is limited, the risk of donor site morbidity and wound infection is high, and the surgical time is drastically prolonged, resulting in the patient’s discomfort [15,16,17]. Through this development, alternative sources of bone grafts have been explored [18]. Allografts originate from the same intraspecies, while xenografts are of bovine or porcine origin. Concerns have been reported that these alternatives have certain drawbacks, including pathogen transmission and immune rejection [15,19]. Another category of bone grafts is synthetic alloplasts, which are fabricated from ceramics, polymers, and metals [4,20]. Even though they are able to withstand increased mechanical load and stress, their utilization is limited. Additionally, their limited integration with the host’s tissue at the defect site, and the considerable risk of infection or failure due to fatigue during implantation, has been reported [15,19,21].

Due to the beforementioned drawbacks of bone grafting materials and the necessity to reconstruct the alveolar bone defects, novel approaches have been investigated. BTE and regenerative medicine (RM) have developed a new concept of utilizing scaffolding nanosystems either alone or combined with growth factors and cell or gene delivery. This concept, termed tissue engineered construct (TEC), may enhance bone repair and regeneration [15,19,20,21,22,23]. The development of a functional TEC from BTE/RM requires: (a) the presence of appropriate cells, (b) a scaffolding material that supports cell growth into an organized tissue, (c) the use of biological factors to promote cellular activity and the formation of bone tissue, and (d) the vascularization of the TEC, which will provide nutritional and oxygen supply for the implanted cells as well as eliminating catabolic end products [24,25]. Additionally, scaffolding materials can be utilized as drug delivery systems, to promote tissue healing and enhance the therapeutic effect through the release of therapeutic agents [26,27,28]. The release of antibacterial agents incorporated inside these scaffolds may suppress bacterial growth and inhibit postoperative infections which are critical in oral and maxillofacial surgery [12]. Thus, Donos et al. (2023) suggested that the mechanical and antibacterial properties of GBR scaffolds should be further explored [12].

According to Walmsley et al. (2015), conventional scaffolding nanomaterials possess poor physicochemical properties and mechanical strength, and low cellular differentiation, while being unable to synthesize the necessary extrinsic factors to positively influence osteogenesis. Several authors reported that the combined action of scaffolds with cells and growth factors may not regenerate adequately the bone defect [25]. This statement is based on the inability to control the degradation of the matrix and the delivery of drug and biological growth factors [24,29,30].

In addition to these biomaterials, the utilization of clinical strategies such as GBR has improved the clinical outcomes of those cases [31]. GBR has become the standard clinical approach technique to restore bone defects, promote bone regeneration, and augment alveolar ridge volume in the oral and maxillofacial region [9]. This approach is frequently used in dental implantology to ensure the long-term prognosis of osseo-integrated implants [2,32]. A variety of materials are utilized in those approaches including bone substitutes and membrane barriers. Membranes are used to selectively promote the adhesion, migration, and proliferation of osteoblasts, while excluding the infiltration of other rapidly proliferating connective and epithelial tissue which would arrest osteogenesis [2,32,33,34]. Bone regeneration is a complex process with various critical factors affecting its initiation such as a source of cells, a scaffold that facilitates bone matrix deposition, signaling molecules, mechanical stability and adequate blood supply [12].

Conventional/monophasic scaffolds were determined to be inadequate to mimic the complex morphology of bone. Thus, the development of multiphasic scaffolds with distinguishable compartments, different biomechanical composition, and tailored architecture that simulates the desired tissue characteristics, may be a promising tool to achieve bone regeneration [6,35]. These multifunctional scaffolds are osteoconductive, can act as barriers, release bioactive substances, and consequently promote bone regeneration, mineralization, and clinical bone repair [36,37]. The progression of technology will eventually address the current limitations in biomaterial fabrication, image acquisition, model development, and design. The advances in image acquisition may improve spatial resolution and accuracy, thus an accurate representation model of the native bone can be developed. The utilization of novel fabrication techniques and the optimization of methodology to control pore shape and size will result in advanced 3D scaffolds development. These systems may possess improved properties, complex architecture, drug/molecular loading capacity, and the ability to direct bone regeneration and healing [6,38,39]. 

This review aims to provide information on the scaffolding systems used to treat alveolar bone defects. Additionally, a variety of manufacturing methods to produce these systems will be described and their role in GBR and dental implant placement will be presented.

## 2. Methodology

This literature review was conducted through different official databases, including PubMed, Google Scholar, Elsevier, and ScienceDirect, to identify the relevant publications according to the topic. Various keywords have been used in those search engines such as “scaffolds”, “nanomaterials”, “alveolar bone defect”, “tissue engineering”, “regenerative medicine”, “alveolar bone regeneration”, “guided bone regeneration”, “dental implants” and combinations of those terms. No limitation regarding an article’s publication date was set, but articles published during the last 6 years were preferred.

## 3. Guided Bone Regeneration Technique and Its Role in Alveolar Bone Defects

Guided bone regeneration is a frequently applied and predictable technique to restore bone defects in the maxillofacial region. Depending on the defect’s morphology and severity of bone loss, concepts such as vertical and horizontal bone regeneration have been widely explored [40,41,42]. These two aspects strongly influence the type, extent, and prognosis of the rehabilitation procedure [43]. A variety of materials are utilized in those approaches to promote the adhesion, migration, and proliferation of osteoblasts [2]. Additionally, polymeric nanomaterials are used as a physical barrier to prevent the ingrowth of rapidly proliferating connective and epithelial tissue at the defect site, thus promoting bone regeneration [34]. The utilization of various grafting materials, including autografts, allografts, and xenografts, has become a common practice for clinicians.

A pivotal clinical approach to address dentition and bone defects is the combination of GBR with dental implants. Frequently, the insufficient alveolar bone volume occurring from local factors including periodontitis, trauma, and localized alveolar process resorption, may be challenging to restore [44]. To overcome this issue, various methods have been employed such as GBR combined with bone grafts and barrier membranes [45]. While autogenous bone grafting may have a limited capacity to restore larger bone defects, the utilization of allografts and xenografts could overcome the challenges in bone augmentation [46]. The barrier membranes’ role is to form a protective layer at the defect site, hindering the ingrowth of proliferating connecting and fibrous tissue. Moreover, they create a microenvironment that would enhance alveolar bone regeneration and provide the necessary bone volume for dental implants [47]. Alternatively, GBR combined with barrier membranes and bone grafts could be used to regenerate bone defects around dental implants (e.g., dehiscence or fenestration defects) [48]. Additionally, several authors have reported that the utilization of membranes are carriers for various growth factors such as bone morphogenetic protein-2 (BMP-2), insulin-like growth factor (IGF), and other factors that promote bone regeneration and development [49,50]. Membranes also provide blood clot stability, nutrient and oxygen transportation, and establishes microcirculation of the treated defect. Omar et al. (2019) stated that membranes are not only hosting but modulating the membrane-associated cellular activities and processes [50]. Thus, choosing the appropriate GBR membrane may have a substantial impact on the therapeutic outcome [44]. The healing of a critical-sized bone defected through GBR without the use of barrier membranes requires scaffolds that are able to support space maintenance, promote bone development, and inhibit fibrous soft tissue ingrowth. Wang et al. (2022) suggested the utilization of osteoconductive bone substitutes, collagen, and poly(lactic-co-glycolic acid) (PLGA), among other materials [2].

### 3.1. Biomaterials for Bone Regeneration

Bone grafting materials are frequently used by clinicians in larger defects to overcome bone’s self-healing limitations. According to the literature, the ideal bone grafting material should possess various key properties including [51,52,53,54]:

- Biocompatibility, a critical property that prevents an inflammatory response;
- Controlled biodegradability;
- Adequate pore size, minimum requirement is 100 μm, but larger than 300 μm is the optimal for vascularization and bone formation;
- Interconnected porosity, which allows the diffusion bone cells, nutrients and waste products;
- Appropriate surface, that allows cell attachment, migration and proliferation while promoting vascular ingrowth;
- Tolerable elasticity and mechanical compressive strength, supporting the adjacent tissue load.

Autografts have been considered the gold standard grafting material for bone repair due to their histocompatibility and non-immunogenic nature. Additionally, they have been characterized as osteo-inductive and osteoconductive, while simultaneously promoting osteogenesis [55]. Despite these benefits, concerns have been made regarding donor site injury, morbidity, and scarring. A patient’s autologous bone can be harvested from a healthy site, which increases the risk of bleeding, inflammation, pain, and infection at the operated sites [54].

Allografts are bone grafting materials harvested from individuals of the same species. Their benefits include the histocompatible nature and the availability in different forms, depending on surgical site requirements [56]. Compared to autografts, concerns have been reported regarding the increased risk of infection transmission and immunoreaction and the high long-term failure rate [54,57,58]. 

Xenografts are bone grafting materials harvested across different species. Concerns have been reported for disease transmission, increased immune response of the host, reduced osteoinductive properties, and the absence of viable cells [59,60]. Even though xenografts have been used in various regenerative approaches with positive clinical outcomes, a higher patient reactivity compared to the other bone grafts have been documented [61].

Alloplasts are synthetic grafting materials developed to overcome the disadvantages of the beforementioned categories, among others, limitations in bone harvesting capacity, immunological reactions, infection transmission, and long-term failure. Various products have been developed such as hydroxyapatite (HA), biphasic calcium sulfate (BCS), β-tricalcium phosphate (β-TCP), and biphasic calcium phosphate (BCP) [62]. These materials are biocompatible and osteoconductive with low production costs [34]. It has been observed that β-TCP has a similar degradation rate compared to new bone formation while providing the in-growth of vascular and cellular components [63,64]. Different research groups reported that the combination of BCS with β-TCP may offer enhanced healing while HA is similar to the inorganic bone matrix [65,66]. β-TCP has been coated with poly(lactic-co-glycolic acid) (PLGA) on alveolar ridge preservation, maintaining the necessary alveolar dimensions and stability requirements for dental implant placement [67].

### 3.2. Membranes (Resorbable/Non-Resorbable)

Guided bone regeneration membranes have been widely used in the field of bone regeneration to isolate alveolar bone defects with a resorbable or non-resorbable mat-like material. The membrane’s role is to function as a physical barrier obstructing gingival cell invasion and proliferating connective tissue ingrowth. These materials should possess a variety of characteristics to positively influence bone regeneration including [68,69,70]:

- Biocompatibility, to integrate with host’s tissues without initiating an inflammatory response;
- Biodegradability, with an appropriate degradation profile according to the host’s tissue;
- Biological activity;
- Competent physical and mechanical properties;
- Porosity and occlusive properties;
- Tolerable strength to withstand the forces of adjacent tissues, preventing membranes collapse;
- Exposure tolerance.

The “gold standard” for non-resorbable barrier membranes are the high-density polytetrafluoroethylene (n-PTFE) and the titanium-reinforced version of high-density PTFE (tn-PTFE). These membranes have been used in GBR and/or GTR procedures due to their exceptional properties, among which is the exclusion of the undesired cells to interfere in the bone healing process, thus facilitating bone regeneration. Due to their non-biodegradable nature, an additional surgery is required for their removal, resulting in patients’ discomfort and pain [71]. Consequently, a variety of natural, synthetic, and composite materials have been developed to replace the non-resorbable membranes with degradable ones [72]. To create a resorbable product, distinctive material-processing techniques based on solvent casting and melting have been utilized to create polymer-based membranes. Additionally, solvent casting/particulate leaching and phase inversion were utilized to create pores on both membranes and 3D scaffolds [71]. Lastly, electrospinning is a promising technique for processing membranes to synthesize biomimetic nanomatrices. Another category of resorbable membranes is synthetic membranes. They are based on polyesters such as PGA, poly(lactic acid) (PLA), and polycaprolactone (PCL) and their co-polymers or tissue-derived collagens. Their favoring properties assisting in the regeneration of periodontal apparatus include biocompatibility and biodegradability (4–6 weeks) with superior handling compared to PTFE membranes [18,71].

## 4. Scaffolds in BTE/RM

The utilization of scaffolds in tissue engineering and regenerative medicine can provide a key element to enhance the regeneration of tissue defects. The biocompatible nature alongside its physicochemical characteristics may reproduce a native extracellular matrix and provide the environmental characteristics to promote cell adhesion, proliferation, and differentiation [25]. According to Saiz et al. (2013) and Hosseinpour et al. (2017), scaffolds are required to modulate the interaction between functions and materials, including cell encapsulation, control drug/chemical release, and scaffold’s engineered surface [24,30]. In order to design an ideal scaffold, it is important to consider the degradation kinetics and physicochemical properties as well as the stimulation of tissue ingrowth, maturation, and remodeling [21,24,73]. 

Various nanocarriers have been evaluated for bone regeneration including polymers of synthetic (PLA, PCL, PGA) or natural (chitosan, alginate, collagen, fibrin) origin, bioceramics/glass (HA, β-TCP), and composites (PLA-chitosan, PLGA-HA) [15]. The incorporation of growth factors and cells in those delivery systems may positively influence the regeneration process through the formation of a microenvironment that resembles the target tissue natural state. A plethora of stem cells such as adipose-derived (ADSCs), mesenchymal (MSCs), induced pluripotent (iPSCs), and bone-marrow stromal cells have been employed from various research groups. Additionally, cell-specific markers and transcription factors have been determined and analyzed including alkaline phosphatase (ALP), osteopontin, osteocalcin, osteonectin, and Runx2. These groups may help to assess the osteogenicity during stem cell differentiation [74].

### 4.1. The Critical Properties of Scaffolds

**Figure 1:**
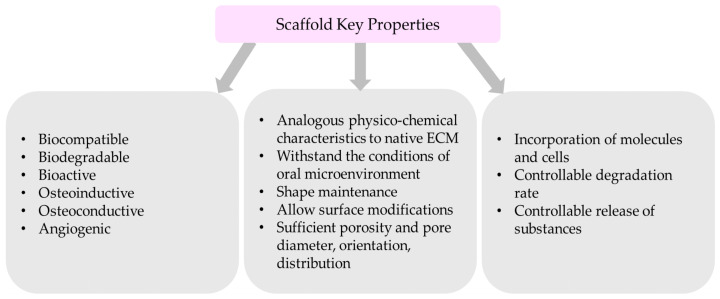
Summary of scaffold key properties.

The regeneration of maxillofacial and oral osseous defects requires the development of a scaffold nanosystem that may mimic the structural, mechanical, chemical, and biological properties of the patient’s bone. Thus, scaffold’s development and design are based on critical considerations that influence the materials used, the fabrication techniques and the functionalization methods [15,24,25,30,55,75,76,77]:

- Biocompatible nanomaterial with non-toxic degradation;
- Bioactivity, which will promote the interaction of a material’s surface and the adjacent cells;
- Analogous physicochemical characteristics similar to extracellular matrix (ECM) of the targeted bone native state;
- Ability to withstand the conditions of oral microenvironment (pH, temperature)
- Shape maintenance after implantation;
- Sufficient porosity and adequate pore diameter, orientation, and distribution
- Allow the incorporation of molecules and cells;
- Allow surface modifications;
- Degradable;
- Controllable degradation and release of substances. Scaffold’s degradation should be similar to the tissue regenerated;
- Osteoinductive and osteoconductive properties to promote cell infiltration;
- Angiogenic;

These ideal properties are summarized in Figure 1.

### 4.2. Scaffold Architecture

The biomimetic scaffold-based approach requires the inclusion of several characteristics to achieve adequate bone regeneration, replication of the missing tissue structure, biological, and mechanical properties. The bone substitutes incorporated into the scaffold should promote osteoinductivity, osteoconductivity, and osseointegration [78]. Osteoinduction is the process of stimulating pluripotent precursor cells to differentiate into osteoblasts [78,79]. Osteocondution promotes the development of the scaffold’s surface as well as within its pores and channels through cell adhesion, proliferation, and eventually forming a new extracellular matrix [80]. Overmann et al. (2020) stated that osseointegration is the establishment of a direct and stable connection between the scaffold and bone tissue without the ingrowth of fibrous tissue [81].

Scaffolds in regenerative strategies are required to be not only biocompatible but also biodegradable to promote the tissue’s innate healing [81,82]. Hence, the optimal system’s degradation rate should be equal to tissue’s regeneration rate. A variety of factors may influence the resorption rate including the local tissue environment, scaffold’s composition, and rate of disintegration. The two main mechanisms responsible for scaffolds resorption are passive hydrolysis (on natural polymeric scaffolds) and enzymatic cleavage (on synthetic polymeric scaffolds). Naturally, these polymers would eventually degrade, but the degradation rate is determined by factors such as molecular weight, comonomer ratio, residual monomer content, chain structure, annealing, crystallinity, and sterilization techniques. Hence, successful tissue regeneration relies on designing a scaffold based on the intricate interplay of those mechanisms while also synchronizing the rates of degradation and tissue regeneration [28,83,84]. Additionally, the capacity of intrinsic variability of patient’s regenerative mechanisms may significantly influence the tissue regrowth. Hence, the material of choice (natural or synthetic polymers, ceramics, and others) may vary in each individual case [85,86]. The plethora of materials utilized as scaffolds possess distinct characteristics regarding elasticity, stiffness, and compressive strength, thus influencing mechanical support and eventually regeneration [78,86,87].

Scaffold’s architecture is crucial, because it provides structural support and orientation to the endogenous and exogenous cells [88]. Moreover, it constitutes the appropriate microenvironment for scaffold-to-tissue integration and cell-to-cell interaction at the site of implantation. Thus, it is pivotal to develop 3D constructs with high porosity and enhanced interconnectivity [53,89]. A key element of bone repair and regeneration is facilitated shortly after implantation with the blood infiltration into the scaffold through the porous structure. Additionally, blood clots are stabilized, and an early microenvironment is formed [90,91]. The pores with a larger diameter ranging between 100 and 700 μm may promote the vascularization process, while smaller ones can inhibit cell growth due to a localized ischemia [53,92,93,94,95]. As mentioned earlier, high porosity is critical to support the transportation of gases, nutrients, and waste product removal. Consequently, the metabolic process and cellular growth is achieved [96,97,98,99].

Extracellular matrix (ECM) is a vital component in nature with an amorphous porous structure. Through bioactive molecules, mechanical stimuli, and spatial patterning, this natural scaffolding system modulates cellular recruitment, growth, and differentiation [100]. In that regard, the decellularized extracellular matrix has been used in tissue repair and regeneration to imitate a 3D microenvironment at the defected sites. Various sources of decellularized ECM are available for clinicians, such as human, bovine, and porcine dermis and human amniotic membrane [101]. Attempts have been made by different research groups to reproduce the hierarchical anatomy of periodontium with biomimetic scaffolds, periodontal progenitor cells, and decellularized ECM [102,103,104,105].

**Figure 2:**
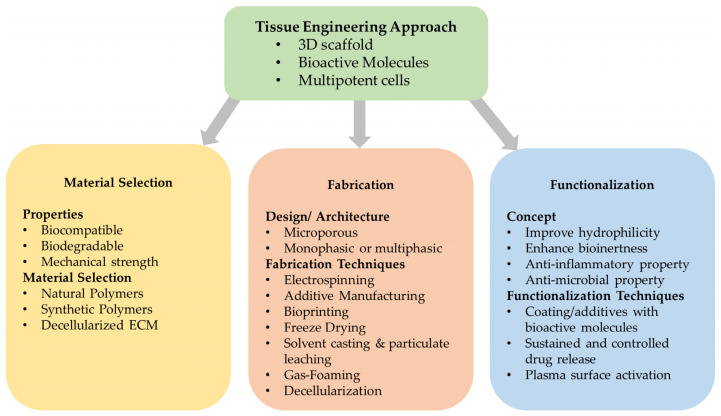
Summary of the various elements influencing scaffold’s design and fabrication concept. In tissue engineering, bioactive molecules and multipotent cells are combined with 3D scaffolds. The scaffold’s characteristics are determined from the materials used, the fabrication techniques and design and the functionalization process. Adapted and modified from Yamada et al. (2022) [76].

Scaffolds are the core of tissue-engineered constructs and may provide cells with the appropriate spatiotemporal guidance through their complex architecture [88]. These characteristics are dependent on a scaffold’s design, material selection, fabrication technique employed, and functionalization method. Figure 2 summarizes the various elements of this tissue engineering approach [76].

### 4.3. Scaffold Fabrication Methods

**Figure 3:**
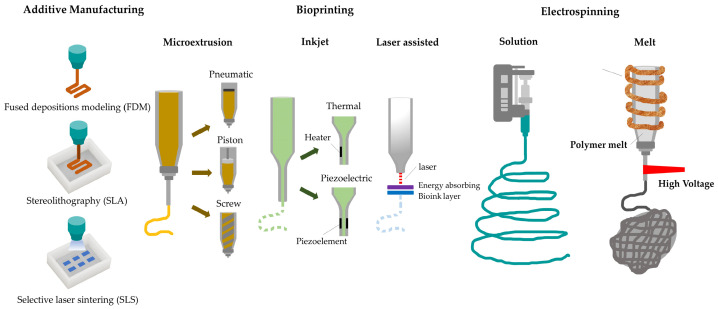
Dentoalveolar scaffold biofabrication approaches. Additive manufacturing: (FDM, SLA, SLS). Bioprinting: microextrusion, inkjet (droplet-on-demand), laser-assisted. Electrospinning: solution-based, melt electrowriting.

Since their early development, a variety of biofabrication techniques have been used to manufacture biodegradable scaffolds and tissue-engineered constructs with highly customizable geometries. The most frequently used approaches are electrospinning and the additive manufacturing technique with the incorporation of relevant cells in a later stage of production. Electrospinning is a technique that can fabricate nanoscaled to microscaled fibrous scaffolds which can mimic the patient’s collagen fibrous network [106,107,108,109,110]. With this fabrication method it is possible to develop highly porous nanoscaffolds with various pore sizes and shapes similar to the native extracellular matrix [111]. Due to the low tunability of the pore sizes, shapes, orientation, and distribution that electrospinning offers, novel approaches that will be later described have been investigated. In that regard, 3D printing may fabricate a multiphasic nanosystem compared to the monophasic nanosystem that electrospinning offers [76,89]. Additive manufacturing techniques could be subdivided into stereolithography (SLA), fused deposition modelling (FDM), digital light processing (DLP), direct ink writing (DIW), and selective laser sintering (SLS) [6]. Gas foaming and salt leaching techniques both use gas and salt, respectively, as porogen additives, in comparison to freeze drying and phase separation techniques that use sublimation and volatilization of solvent and water into the polymer solution [76]. Bioprinting is a biomimetic approach to form tissue-engineered constructs which combines hydrogels and cells [112]. Bioassembly is another technique that has been reported but has a limited use for dento-alveolar regeneration [113,114]. The most popular biofabrication approaches to develop scaffolds for dentoalveolar regeneration are additive manufacturing, bioprinting, and electrospinning. These biofabrication approaches and their variations are illustrated in Figure 3. 

#### 4.3.1. Electrospinning

This technique applies high voltage to a polymeric solution to create a nanofibrous or microfibrous scaffold [115]. The high voltage overcomes the liquid’s surface tension resulting in the elongation of liquid droplets to nanofibers. An electrospinning apparatus is comprising [116,117,118]:

- High voltage power supply;
- Syringe pump;
- Metallic needle;
- Stationary or rotating metallic collector for fiber collection.

The scaffold is formed when the fiber collector and spinneret are connected to electrical terminals with opposite ends. The material is drawn out from the potential difference and deposited onto the collector fabricating the desired nanofibers [119]. Electrospinning may produce highly porous polymer structures of natural or synthetic origin with an increased surface area such as gelatin nanofibers, collagen and polycaprolactone (PCL) [120]. Several research groups have used electrospinning to fabricate scaffolds for alveolar bone regeneration, implant integration, gingival tissue and periodontal ligament regeneration [121,122,123,124,125,126,127]. This technique produces meshes with an increased surface area and high porosity promoting cell attachment. Small pore size and densification may hinder cell migration. The development of a novel solution-based electrospinning apparatus enhanced fiber deposition control, and consequently, the fabrication of microporous meshes and pre-designed struts was achieved [128]. Electrospun meshes have been used for controlled drug and molecular release. According to Rad et al. (2019), they are not only effective in promoting faster regeneration, but they can also suppress bacterial colonization through the incorporation of bioactive glass nanoparticles [129].

In comparison to solution electrospinning, melt electrospinning may adequately control fiber deposition. The pore size of the fabricated structure is above micron size. Recently, two research groups have reported the combination of FDM and electrospun melt meshes of PCL origin to produce a biphasic scaffold [130,131].

#### 4.3.2. Additive Manufacturing

Additive manufacturing (AM), also known as 3D printing, incorporates a group of novel techniques used to fabricate three-dimensional (3D) tissue-engineering constructs that have been designed through computer-aided technologies in a layer-by-layer approach [132,133]. The bone defect is scanned with magnetic resonance imaging (MRI), or cone beam computed tomography (CBCT) and a scaffold model with volumetric shape that would fit into the defect is then designed [132,133,134,135]. The most abundantly used AM techniques in dentoalveolar settings are SLA, SLS, and FDM [46]. Selective laser sintering of ceramics, polymers and their combination, and selective laser melting (SLM) techniques have been used to form 3D constructs layer by layer. Various groups have applied these scaffolds in alveolar bone augmentation [136,137,138]. A patient-specific implant has been developed by Rasperini et al. (2015), utilizing PCL and SLS. According to the authors, limited regeneration was observed due to the polymer selected and scaffold’s design [137]. Through fused-deposition modeling, thermoplastic polymers or composite polymers can be processed with inorganic materials. The applicability of this technique has been reported in alveolar bone augmentation, periodontitis treatment, and whole tooth regeneration [127,130,137,139].

#### 4.3.3. Bioprinting

Bioprinting could be classified as an additive manufacturing process due to their similarities, and Direct Ink Writing (DIW) can enable the development of complex 3D structures for biomedical applications [140]. Through this approach, developers have combined hydrogels and cells to fabricate biomimetic tissue-engineered constructs [112]. The most abundantly used bioprinting techniques are light or laser-based, extrusion-based and inkjet or droplet-on-demand [112]. These technologies utilize a variety of bioinks and particularly cells, hydrogels, or their combination [141]. The 3D printers utilized in the biomedical field may accurately fabricate scaffolds with a resolution of 10 μm or more [142,143,144]. The lowest acceptable limit of pore diameter that significantly promotes osteogenesis has been defined by Hulbert et al. (1970) at 100 μm [145]. The larger diameter pores (150–200 μm) have been reported to facilitate the highest degree of new bone formation, which is within the range of the Haversian bone system (100–200 μm) [146]. Additionally, smaller pore sizes (less than 100 μm) can promote chondrogenesis before osteogenesis, while low porosity with non-interconnected pores may prevent nutrient transportation. Hence, bone regeneration is hindered [147,148,149]. Different research groups have used extrusion-based bioprinters and combined them with bioinks to produce dental constructs [141,150,151]. Droplet-on-demand (DoD) is a bioprinting technique that can fabricate only small scaffolds. This limitation is related to the frequency used, droplet volume, and the actuation mechanism [152]. Laser-based bioprinting delivers small volumes of bioinks to the targeted platforms through a laser beam. Few authors have reported its use for the development of dento-alveolar constructs [153,154,155,156].

#### 4.3.4. Freeze Drying

Freeze drying is a three-step process of drying polymeric solutions. It starts with solution preparation, followed by molding or casting of the solution, and later, freezing and drying under low pressure. At the last stage of fabrication, the ice and water are removed through sublimation and desorption, respectively. The scaffold’s pores may range between 15 and 200 μm with up to 90% porosity. Pore size can be modulated through temperature, polymer concentration, and freeze rate [157]. The utilization of a high-strong vacuum is mandatory to fabricate scaffolds with interconnectivity and increased porosity [6]. Natural or synthetic polymers and composites can be fabricated through this process including gelatin/hyaluronic acid and collagen/hyaluronic acid [6,158]. Shrestha et al. (2021) developed an artificial bone extracellular matrix substitute with favoring biological behavior and excellent osteoinductive properties. According to the authors, this system of multiwalled carbon nanotubes incorporating zein and chitosan into polyurethane may ensure bone cell regeneration and can be used as an artificial bone-grafting material [159].

#### 4.3.5. Solvent-Casting and Particulate Leaching

This is a commonly applied technique to develop scaffolds when mixing water-soluble salt particles such as sodium citrate and sodium chloride into a biodegradable polymer solution. In order to remove the solvent, a process called lyophilization is applied to the mixture. Leaching out the salt particles will result in the development of a porous scaffold. This approach is simple and provides adequate control of the pore size and porosity. The variability of salt’s particle size and salt-to-polymer ratio will strongly influence the scaffold’s structure [160]. Through this fabrication technique a PLA/HA composite scaffold has been developed by Zimina et al. (2020) with 79% porosity, good suppression of tissue ingrowth, and improved adhesion of the mesenchymal stromal cells compared to the PLA alone. Additionally, due to the addition of HA, the system presented a limited inflammatory response and has been suggested for the restoration of maxillofacial defects [161].

#### 4.3.6. Gas-Foaming Process

Gas-foaming process is a scaffold fabrication technique that can be classified into chemical foaming and physical foaming [162]. This characterization is based on the development of a blowing agent into the polymeric matrix. In tissue engineering and regenerative medicine, chemical foaming is prohibited due to the residues inside the polymeric matrix that may influence the scaffold’s biocompatibility [163]. Alternatively, physical foaming utilizes blowing agents, including N_2_ and CO_2_, at a high pressure to saturate the polymer disks [164]. This high pressure is later reduced, resulting in a thermodynamic instability and the formation of a 3D porous polymer structure is achieved [163,164]. Scaffolds with a pore size of approximately 100μm and up to 93% porosity but with poor interconnectivity could be fabricated through this method [164,165]. Sukpaita et al. (2021) investigated the mineralized tissue regenerative potential of a biocompatible, biodegradable, osteoconductive, and chitosan-based scaffold. Despite its advantages, the authors concluded that pure chitosan is not adequate to support regeneration due to its limitations such as rapid degradation rate, poor mechanical properties, and low osteoinductivity. Thus, chitosan should be combined with other biomaterials and/or bioactive molecules to improve the system’s characteristics [166]. 

#### 4.3.7. Decellularization

Decellularization is the process of cell removal while preserving key properties of extracellular matrix such as architectural integrity and composition. Additionally, the decellularized ECM should be able to promote cell growth and differentiation as before. The plethora of processing techniques used to obtain the decellularized bone matrix include enzymatic methods, surfactants, hydrostatic pressure, thermal shock, and sonication [167]. Hydrostatic pressure is a promising technique that minimizes protein denaturation while also preventing the use of chemical agents, hence providing a higher quantity of ECM [168]. The last step of decellularization involves the incorporation of dehydrated alcohol and nucleases to eliminate cellular remains. In bone tissue engineering, decellularized bone matrix has been frequently employed as a scaffolding system to mimic native bone [6,169]. Santos et al. (2024) investigated the regenerative properties of an electrospun PCL/Chitosan nanofibrous scaffold loaded with bioactive cell-derived extracellular matrix. This nanosystem promoted cell proliferation and enhanced osteogenic differentiation, while also increasing bone-specific marker gene expression, calcium deposition, and alkaline phosphatase activity. Additionally, higher cell mineralization was observed and the scaffold’s use for alveolar bone regeneration was suggested [170]. 

The advantages and disadvantages of those techniques are summarized in the following table (Table 1), along with material examples fabricated through those methods. 

### 4.4. Biodegradable and Nonbiodegradable Scaffolds

The materials used for a scaffold may be either biodegradable or non-biodegradable. Biodegradable scaffolds allow tissue neogenesis through the replacement of the degraded biomaterial. These materials can be classified as bioactive, biotolerant, and bioinert, depending on the implanted tissue [188]. The novel scaffold fabrication approaches require modern biomaterials that can interact with the stem cells incorporated inside the scaffold and promote their differentiation. Both scaffold categories are able to direct and regulate stem cell differentiation into the desired somatic cells [189]. Biomaterials of natural origin (e.g., collagen, elastin, fibrin, alginate, chitosan) are biocompatible and biodegradable with a low immune response. Their limitations could be summarized as poor mechanical strength, inconsistent purity resulting in lot-to-lot variability, and difficulty in sterilization and purification [189,190,191]. 

Synthetic biomaterials are of non-natural origin and can be produced at a large scale. Additionally, they can have tunable characteristics such as high flexibility, controlled composition and degradation rate, and improved mechanical properties while allowing their functionalization [189]. The most frequently used biomaterials, among others, are polycaprolactone (PCL), poly(glycolic acid) (PGA), poly(lactic-co-glycolic acid) (PLGA), polyvinyl alcohol (PVA), and poly(ethylene glycol)diacrylate (PEGDA) [77,189].

### 4.5. Additional Scaffold Categories

#### 4.5.1. Monophasic Scaffolds

The first development of monophasic scaffolds (MNPS) was inspired by the concepts of GBR/GTR. It was based on the utilization of biomaterials for space maintenance and to promote tissue neogenesis [192]. Consequently, monophasic tissue engineered constructs were fabricated following those principles. To overcome their impaired bioactivity, they were combined with biological additives and fillers. A well-documented approach to enhance the regenerative effect of those scaffolds was the encapsulation of cells in hydrogel systems or their placement directly into the scaffold, which was consecutively transplanted into the defect site. In this approach the MNPS were not only a nanoplatform for targeted cell delivery but also a barrier to maintain space for cell growth [35]. Alveolar bone regeneration facilitated by MNPS was determined to be not sufficient to restore the defect site at its primary condition. Consequently, the development of multiphasic scaffolds was apparent [193].

#### 4.5.2. Multiphasic Scaffolds

**Figure 4:**
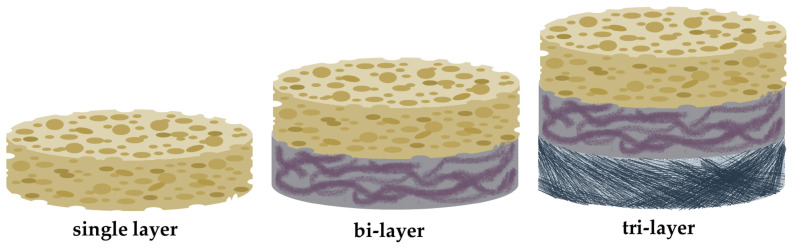
Illustration of monophasic scaffolds (single layer) in comparison to the distinguishable compartments of multiphasic scaffolds (biphasic-bilayer and triphasic-tri-layer) that requires different materials, fabrication techniques, and functionalization approaches.

The development of multiphasic tissue-engineered constructs was necessary to achieve periodontal regeneration. These scaffolds have distinguishable compartments with different biomechanical composition and architectural nature, such as pore size/shape and porosity. These constructs can even mimic the strict hierarchical organization of periodontium. Multiphasic scaffolds are a large group of tissue-engineered constructs consisting of biphasic and triphasic scaffolds [35]. Their distinct compartment design is illustrated in Figure 4 and is compared to monophasic scaffolds. A variety of groups have developed biphasic scaffolds to achieve periodontal ligament and alveolar bone regeneration, but their attempts failed to provide a new cementum layer at the interface of the tooth. Their approach was based on the ability of endogenous cells to promote new cementum apposition, or the utilization of in vitro differentiated cells. Thus, the development of multiphasic scaffolds incorporating a third layer, which will promote cementogenesis, is necessary [35]. Triphasic scaffolds have emerged to address the issue of cementum regeneration. The three-compartment design may promote the formation of cementum on the root surface, direct the insertion of the periodontal ligament, and provide adequate rigidity [194]. Designing such a complex biomaterial may be challenging, while also the presence of low interphase cohesion resulted in a diminished mechanical stability of the system. The utilization of various novel fabrication techniques including simultaneous multiphase crosslinking and additive fabrication may address this dilemma [195,196].

#### 4.5.3. Hybrid Scaffolds

Hybrid scaffolds are technological products that are developed to regenerate multi-tissues (e.g., alveolar bone and periodontal ligament) through the combination of scaffolds with various geometrical scales [46,130,197]. Vaquette et al. (2021) combined FDM with melt electrospun scaffolds in a multidimensional hybrid alveolar bone augmentation approach [130]. The melted electrospun mesh was incorporated into the core of scaffolds and the latter were surrounded by FDM constructs. Poly-l-lactic acid was used to cover the scaffolds in all sides except one, which was in contact with the bone. According to the authors, at 8 weeks, a sufficient bone had been formed, and after the protective case’s removal, a dental implant was placed [130].

#### 4.5.4. Smart Scaffolds

Various smart biomaterials have been developed to enhance tissue repair and regeneration. These materials possess intelligent characteristics and functions [198]. The development of a smart construct requires the incorporation of bioactive materials with tunable physicochemical characteristics [199,200].

Categories of smart scaffold constructs combined with stem cells for bone tissue engineering include the following:

- Biomimetic and bionic. Mittal et al. (2010) developed a porous biomimetic scaffold containing PLGA microspheres and peptides. This system was able to replicate the structure and composition of natural tissues [201].
- Immune sensitives. Zeng et al. (2017) coated a mesoporous bioactive glass scaffold with amino functional groups and reported its osteoimmunomodulatory efficacy on MSCs, macrophages, and bone marrow [202].
- Shape memory. Liu et al. (2014) loaded a shape-memory nanoporous scaffold with growth factors (BMP-2), attempting to repair a mandibular bone defect. The authors stated that the nanosystem could be applied in bone-regenerative medicine due to its potential [203].
- Electromechanical stimulus. Damaraju et al. (2017) developed flexible 3D fibrous scaffolds that are able to initiate the differentiation of MSCs and tissue formation [204]. A similar scaffold (Piezoelectric poly(vinylidene fluoride-trifluoroethylene)) incorporating zinc oxide nanoparticle enhanced the adhesion and proliferation of hMSCs while also improving blood vessel formation [205].

#### 4.5.5. Personalized Scaffolds CAD/CAM

**Figure 5:**
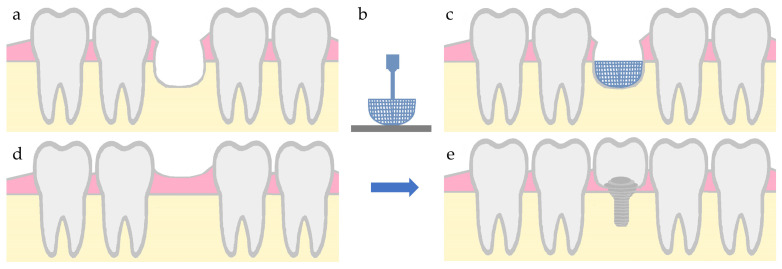
Illustration of an alveolar bone defect regeneration with a printed scaffold, followed by dental implant placement. (**a**) defect in bone tissue, (**b**) additive manufacturing of a bone scaffold, (**c**) scaffold placement in the defect area, (**d**) bone regeneration, and (**e**) dental implant placement.

Through the progression of technology, it is now possible to achieve personalized fabrication of biomedical tools according to the anatomical defects. The combined action of precise image acquisition and 3D bioprinting equipment may develop individual-specific scaffolds with tailor-made characteristics [206]. This process starts with image acquisition, followed by image processing, 3D reconstruction, and 3D computer-aided design (CAD) modeling. In the last step of fabrication process, termed rapid prototyping, the customized tissue-engineered construct is bioprinted [207]. Then, the personalized bioprinted scaffold could be implanted at the defect site, as shown in Figure 5 [208].

Magnetic resonance imaging (MRI) and computerized tomography (CT) are valuable tools to obtain precise data for the fabrication of a personalized scaffold. Scaffold’s production is achieved through computer-aided design and computer-aided manufacturing (CAD/CAM). Its design and architecture can be modified at a macro, micro, and even nanoscale [75]. The scaffold’s shape, internal porosity, and load-bearing capabilities are obtained based on a patient’s imaging data. When used as drug delivery systems, a microporous structure is necessary to support adequate drug delivery. Moreover, its mechanical properties should be similar to those of native tissues [207,209,210].

### 4.6. Scaffolds as Drug Delivery Systems

Another interesting utilization of scaffolds in regenerative medicine is the controlled drug/molecular release and immunomodulation. This approach may provide new aspects in bone regeneration due to the knowledge acquired at the cellular and gene level. Scaffolds are not only able to support tissue growth and targeted tissue response, but also to prevent undesirable cellular mechanisms. The advancements of scaffold design may improve cellular connection and activation, hence regulating cellular activity. In particular, the inhibition of cell attachment and the activation of cells may assist in the prevention of undesirable biological responses [211].

Zielińska et al. (2023) stated that drug/tissue delivery scaffolds should choose the appropriate scaffold type in order to influence the material’s architecture and structure (e.g., hydrogels, nanofibers, nanopatterns, microparticles, nanoparticles, or matrix) [212]. Additionally, the selection and incorporation of bioactive agents (among other cells, proteins, peptides, pharmaceutical molecules) into the scaffold may improve its characteristics. This process is called functionalization. Moreover, the scaffold’s surface may be further modified and the appropriate drug release profile (sustained, rapid or sequential) should be chosen [212]. The co-delivery of distinctive bioactive agents and drugs can reduce toxicity, minimize the drug dissociation rate, and eventually increase a drug’s effectiveness. Hence, the conceptualization of a scaffold incorporating bioactive agents may provide a universal drug delivery platform [212]. Therefore, they can be utilized as antimicrobial and anti-inflammatory agents when combined with the appropriate drug molecule.

#### 4.6.1. Antimicrobial Effect

The unique structure of scaffolds suggested their use as drug delivery nanoplatforms against periopathogens. Several attempts have been made to ablate periodontal infection by loading antibacterial drugs into scaffolds and then implanting them at the defect site. Ferreira et al. (2021) reported the incorporation of metronidazole and tetracycline in polymeric scaffolds for the treatment of periodontal defects. The authors claimed that the development of a defect-specific antibiotic-laden scaffold was able to sustain periodontal reconstruction while ablating the infection present [213]. Ribeiro et al. (2020) developed a hybrid system of injectable hydrogels loaded with ciprofloxacin with a significant antimicrobial effect against *E. faecalis* [214].

#### 4.6.2. Anti-Inflammatory Effect

Apart from bacterial growth inhibition, attempts have been made to modulate inflammation through scaffolds loaded with pharmacological agents (e.g., non-steroidal anti-inflammatory drugs) [215]. Scaffolds are implanted into the defect sites after the thorough elimination of dental plaque, calculus, irritants, and granulated tissue is performed, thus minimizing the inflammation modulation required after treatment [214]. During wound healing, a mild degree of inflammation at the treated site is expected. On the contrary, persistent inflammation will negatively influence the healing site and treatment outcome [216,217]. Yar et al. (2016) and Xu et al. (2019) reported the use of chitosan-based scaffolds loaded with meloxicam and aspirin, respectively, and both groups concluded that the sustained drug release reduced post-treatment inflammation [218,219]. Comparable results were reported by Batool et al. (2018), in a study determining the anti-inflammatory efficiency of PCL scaffolds loaded with ibuprofen [220].

## 5. Conclusions and Future Perspectives

Both conventional and novel state-of-the-art tissue engineering approaches have proven their potential in bone tissue engineering. The fabrication of scaffolds with characteristics similar to the target tissue such as biocompatibility, structural stability, osteoinductivity, osteoconductivity, physicochemical properties, porosity, adequate pore size, and pore interconnectivity is still a challenge to achieve. The advances of nanotechnology combined with key characteristics on fabrication methodology may assist in the development of novel biomaterials [221,222,223]. These biomaterials will be able to mimic the hierarchical organization and structure of native bone [6]. 

It has been reported that monophasic scaffolds are difficult to meet the requirements to achieve adequate bone regeneration due to the complexity of natural tissue. Scaffold’s architecture is crucial, since porosity, pore size, orientation, and interconnectivity may influence mechanical and biological properties of bone regeneration. Hence, the lack of control of those features during the manufacturing process will negatively impact the therapeutic outcome, especially in larger bone defects. It is apparent that scaffold fabrication techniques should provide tissue-engineering constructs that have a tailor-made structure and a defined pore shape and size. Thus, their advancements should focus on controlling these aspects [6]. Additionally, the ideal tissue-engineering construct should support long-term stability and space maintenance with controllable degradation rate. These characteristics may allow bone remodeling and eventually ensure implant longevity through the prevention of bone resorption upon implant placement [41]. Novel tissue-engineering approaches are utilizing various categories of stem cells, growth factors, genes, and biologic agents to achieve tissue regeneration. These groups may promote, among other bone regeneration, tissue vascularization and wound healing. Hence, it is crucial to understand their mechanisms and identify the appropriate group for each individual case. 

The advances in imaging data acquisition (CT, MRI) and digital design and manufacturing (CAD/CAM) have provided a novel personalized approach in bone regeneration. The bioprinters used are able to produce accurate 3D scaffolds with complex architectures, even though limitations in hardware and materials have been reported. Even though extrusion-based additive manufacturing techniques are able to replicate complex geometries, their poor resolution and slow manufacturing process needs to be adjusted. On the other hand, techniques such as SLA could overcome these limitations, but novel materials with better characteristics should be developed [6]. The fabrication of layered scaffolds through conventional methods including electrospinning, solvent casting, gas-foaming, and freeze drying may have benefits regarding high porosity, good pore interconnectivity, and low production costs, but the complexity of alveolar bone and periodontium requires novel fabrication techniques [224].

Targeted drug delivery has gained more attention during the last decade. The development of advanced tissue engineering constructs—scaffolds—that ensure that proper transport of the incorporated substance and controlled drug release can be a promising tool. In that regard, researchers should enhance the stability and functionality of these systems in order to achieve an improved therapeutic outcome [212].

## Figures and Tables

**Table 1:** Advantages and disadvantages of scaffold fabrication methods.

Fabrication Technique	Material Examples	Advantages	Disadvantages	Reference
Fused Deposition Modeling (FDM)	Thermoplastic polymers and their composites (PCL-TCP scaffold, PCL/poly(glycolic acid) (PGA))	Low cost, simple to use, various lay-down patterns, good mechanical and thermal properties, high porosity, can control porosity and pore size, pore interconnectivity, macro shape control, solvent-free.	High processing temperature, inconsistency in pores, limited application just on PLA and PCL due to the required thermoplastic, materials in filament form, smooth surface, requires support structures for irregular shapes, pore occlusion at boundaries.	[171,172,173,174,175,176]
Direct ink writing (DIW) or microextrusion	Natural or synthetic based Hydrogels (alginate, chitosan, polyethylene glycol (PEG)) Bioceramics	Quick printing speed, low production cost, simple to use/operate, wide range of application, cells embedded into hydrogels, cellular and acellular printing.	Low printing accuracy compared to SLA, resolution approximately 100 μm.	[28,133,177]
Stereolithography (SLA)	poly(ethylene furandicarboxylate) (PEF), PCL	Highest resolution, enhanced versatility, fast speed of production, 5–300 μm accuracy and the smoothest surface finish among the other available techniques, complex 3D structures that may incorporate cells and bioactive agents, through heating it is easy to remove the photopolymer.	Photopolymerization of materials (it can be processed only into photo-crosslinked hydrogels and can be modified by adding photo-crosslinked groups), photocurable, high production and equipment cost, limited range of photosensitive materials.	[28,172,173,175]
Selective laser sintering (SLS)	Ceramics, Polymers (TCP, Hydroxyapatite (HA), PCL) Composites	Fabrication of highly detailed products with thin walls, complex structures with good mechanical strength, can control pore size and porosity independently, high porosity, solvent free, wide variety of materials can be used with the addition of any secondary binder system.	One of the poorest dimensional accuracies (150–180 μm) compared to the other AM fabrication methods, small pore size, unable to incorporate cells and growth factors during printing process, the thermal distortion shrinks and warps the produced scaffold, inability to use natural polymers due to the high temperatures generated by the laser beam, only thermally stable polymers can be used, materials in powder form, difficult to remove trapped materials.	[28,172,173,175,176]
Electrospinning	PLGA/PCL PCL/PEG Silk fibroin	The development of nanofibrous scaffolds is achieved through this technique, fiber homogenous mixture with high tensile strength, simple to use, cost efficient compared to other methods, continuous process, scalability, controllable fiber diameter from nm to microns.	Toxicity of solvents, packaging–shipping handling, jet instability.	[175,178,179,180]
Freeze Drying	Computer-aided design and computer-aided manufacturing (CAD/CAM) bone grafting PTFE/PVA polymers with/without graphene oxide nanoparticles	Solid porogen is not required, highly porous structures with enhanced interconnectivity, control pore size by altering the freezing method, capability to prevent high temperatures.	Organic solvents, limited to small pore size (15–35 μm), irregular porosity, long processing time, high energy consumption.	[171,175,178,181]
Solvent Casting-Particulate Leaching	PLA/HA scaffolds	Simple technique, scaffolds with regular to high porosity (50–90%), controlled pore size and composition, crystallinity can be tailored, low production cost.	The incorporation of biomolecules and cells into scaffolds is hindered due to the organic solvents used, difficulty to adequately control pore shape and interconnectivity, limited mechanical properties and thickness of structures developed, residual porogens and problems with residual solvent, widespread use of toxic solvents.	[161,171,175,182]
Gas-Foaming	Chitosan-based scaffolds	Chemical solvents are not required, and their use is prevented, porosity up to 85%, pore size between 30 and 700 μm, low production cost.	High pressurized technique that prohibits the incorporation of bioactive agents and cells into the scaffolds, difficult to control pore sizes and ensure their interconnectivity, low interconnectivity insufficient mechanical strength the denaturation of materials due to high temperatures during compression molding step can be observed.	[164,166,175,178]
Bioprinting	PCL and alginate Alginate, PCL/alginate mesh Collagen type I/bone dECM/ β-TCP	Low production cost, high degree of accuracy, great shape complexity, high printing speed, capability to support parallel high cell viability.	Depending on the existence of cells.	[179,183,184,185,186,187]

## Data Availability

Not applicable.
